# Reaction time and working memory in gamers and non-gamers

**DOI:** 10.1038/s41598-022-10986-3

**Published:** 2022-04-26

**Authors:** Gal Ziv, Ronnie Lidor, Oron Levin

**Affiliations:** 1 Motor Behavior Laboratory, The Academic College at Wingate, 4290200 Netanya, Israel; 2 Movement Control and Neuroplasticity Research Group, KU Leuven, Leuven, Belgium

**Keywords:** Psychology, Human behaviour

## Abstract

The purpose of this pre-registered study was to examine whether asking gamers and non-gamers about their video game playing habits before or after they performed computerized cognitive-motor tasks affects their performance of those tasks. We recruited 187 participants from an online participants’ recruitment platform. Out of those participants, 131 matched our criteria as gamers or non-gamers. They were then divided to two subgroups, and performed a choice-RT task, a Simon task, an alternate task-switching task, and a digit span memory task either before or after answering a video-game playing habits questionnaire. The results showed that gamers who completed a video-games questionnaire before performing the tasks had faster reaction times (RTs) in the Simon task compared with gamers who answered the questionnaire after performing the tasks. In contrast, non-gamers who answered the questionnaire before the task had slower RTs in the Simon task and the alternate task-switching task compared with non-gamers who answered the questionnaire after performing the tasks. The results suggest that answering a video-games questionnaire before the start of a study can lead to a response expectancy effect—positive for gamers and negative for non-gamers. This may bias findings of studies examining video games and the performance of cognitive-motor tasks.

## Introduction

With over 2.7 billion gamers worldwide^1^, playing video games can be considered as one of today's favorite pastimes. As the popularity of video games grows, research interest in the effects of playing video games on human behavior and psychology increases as well. In the past few decades, researchers have examined the relationship between video games and aggression (e.g.,^2,3^), depression (e.g.,^4,5^), addiction (e.g.,^6^), and cognitive processes—among them executive function (e.g.,^7,8^), attention (e.g.,^9^), reaction time (RT) (e.g.,^10^), and working memory (e.g.,^11^). It has been suggested that playing video games can have cognitive, motivational, emotional, and social benefits^12^.

A number of studies have shown positive relationships between playing video games and cognitive-motor skills (e.g.,^13–17^). For example, Boot et al.^13^ showed that expert gamers are better than non-gamers in tracking moving objects, in detecting change, and in task switching. Colzato et al.^15^ reported that experienced gamers who play first-person shooter games—action games that are played from a first-person view—were more accurate at an N-back task and reacted faster to go signals in a stop-signal task without compromising stopping performance, than non-gamers. Another study^17^ used a stop-change paradigm (a variation of the stop-signal task with the addition of a cue to not only inhibit a response but to initiate another) and demonstrated that, compared with non-gamers, experienced first-person shooter players reacted faster in the go condition and in the change conditions without compromising accuracy.

While the abovementioned findings are promising, there are a number of methodological concerns that undermine our ability to show a causal relationship between playing video games and improved cognitive and motor performance. It is not clear, for example, whether the relationship between gaming and performance is caused by the gaming experience or if it represents pre-existing differences that lead to a self-selection effect, causing certain individuals to choose to play video games^13^. Boot et al.^18^ suggested that several methodological shortcomings may undermine the positive effects of playing video games on cognitive/motor performance. Specifically, for studies that aim at examining differences between gamers and non-gamers, covert recruiting of participants is of importance.

Boot et al.^18^ emphasize that gamers should not know that they are recruited for a study about gamers or about the benefits of playing video games, as this might bias the results. To prevent that bias from occurring, researchers should not ask participants about their video game-playing experience before the study. This methodological argument is supported by the concept of psychological suggestion. Psychological suggestion refers to a process by which individuals or environmental cues influence the way we think and behave^19^. Suggestions can be deliberate (e.g., directly influencing one's thought, beliefs, or behaviors), or unintentional (e.g., given by certain cues given by individuals or that are present in the environment). Examples of such unintentional cues can be found in various domains. For example, jurors' verdicts are affected by judges’ expectations of guilt and by subtle differences in the way they give instructions to the jury^20^. Ziv and colleagues^21^ provided an example from the motor learning domain, where participants’ expectancies of success were manipulated by changing the task-success criterion. In their study, participants who practiced with an easy success criterion putted golf balls more accurately in a transfer task compared with participants who practiced with a difficult success criterion. In the abovementioned studies, subtle cues led to changes in decision making in jurors as well as in individuals who performed a motor task (golf putting). Similarly, gamers who learn that they are about to participate in a study on gamers' abilities, and who believe that gaming may be related to higher cognitive and motor performance, might expect to perform better—and indeed do so.

One theory that can explain how psychological suggestion works—and in the context of the current study how unintentional psychological suggestion can lead to changes in gamers' task performance, is the response expectancy theory^22,23^. Response expectancies can be defined as “the anticipation of automatic, subjective, and behavioral responses to particular situational cues” (^23^, p. 69), and they can be a product of suggestion. Such response expectancies can lead individuals to automatically change their behavior in accordance with their expectancies ^24^. For example, Clifasefi et al.^25^ showed that telling participants that they are receiving a drug that enhances mental alertness and cognitive function, when they actually received a placebo, led to improved performance in a cognitive task (compared with participants who were told they were given a placebo). Similarly, Foroughi et al.^26^ showed that individuals who were recruited overtly for a cognitive training session (i.e., recruitment flyer mentioned that training can improve cognitive function) improved their cognitive performance after a one-hour training session compared with participants who were recruited covertly (i.e., neutral recruitment flyer) for the same training and showed no improvements.

In accordance with psychological suggestion and response expectancy theory, Boot et al.^27^ suggest that participants' expectancies can affect the results of studies. Gamers, for example, may expect to perform well in certain cognitive/motor tasks if they believe that there is a positive relationship between gaming and performance, and if they are aware of the fact that they were recruited for a certain study because they play video games. Such expectancy effects can also occur in video game training studies in which participants are told that such training should lead to improved performance in various cognitive tasks (e.g.,^28^).

To assess the effects of this possible bias directly, we devised a study in which a group of gamers and a group of non-gamers were covertly recruited and were asked to perform certain cognitive/motor tasks either before or after answering a video-games questionnaire. Covert recruitment can be accomplished, for example, by inserting the questions regarding gaming habits within various unrelated questions (e.g., questions about religious beliefs and preferred temperatures)^17^. However, it is even better to avoid asking such questions at all. In our study, we used an online participant recruitment platform that allowed us to recruit gamers and non-gamers without asking any preliminary questions.

Therefore, the purpose of the current study was to examine whether asking participants about their gaming experience prior to participation in the study affects their performance. We hypothesized that (a) asking gamers about their gaming experience before the study will lead to better performance in reaction time (RT)-based tasks compared to asking the same questions after the study; (b) asking non-gamers about their gaming experience before the study compared to after the study will not affect their performance in RT-based tasks; and (c) there will be no differences between gamers and non-gamers in a digit-span memory task.

The second hypothesis requires an explanation. First, although response expectancies and suggestions can be both positive and negative, there are relatively little data regarding these effects in simple cognitive-motor tasks. In addition, the few studies that examined these effects on motor performance showed contrasting results. While Ziv et al.^21^ showed positive, not negative, effects in a golf-putting task, Fillmore and Vogel-Sprott^29^ reported both negative and positive changes in the performance of a pursuit-rotor task corresponding to suggestions of negative or positive effects of caffeine (when the participants actually drank a decaffeinated drink). In addition, Harrell and Juliano^30^ showed the opposite effect of placebo caffeine in a finger-tapping task (improved performance when told caffeine impairs performance, reduced performance when told caffeine enhances performance). Finally, we did not know whether non-gamers believe that gaming is related to performance of such tasks, or if such beliefs are necessary for the effect to occur. Therefore, we adopted a cautious approach in developing this hypothesis. The third hypothesis is based on the view that, as compared with attention and information processing capacity, working memory capacity is expected to be affected to a lesser extent by suggestions or response expectancy (in the context of the beliefs of gamers). Indeed, Boot et al.^27^ have reported that expectancies that playing video games will improve memory stores are relatively low.

We selected RT-based tasks because these tasks are expected to produce better processing speeds, attentional control, and visuomotor transformation, which appear to be more elevated in gamers than non-gamers. Working memory, on the other hand, may be positively affected by gaming to a lesser extent, albeit some improvement might be expected as the memory network and the attentional network share overlapping neural pathways (e.g., dorsal attentional pathways^31^.

### Pre-registration and raw data repository

The study’s main questions and hypotheses, experimental conditions and groups, and dependent variables, as well as the handling of outliers and data exclusion, sample size, and statistical analyses, were all pre-registered on aspredicted.org and can be accessed online (https://aspredicted.org/wp53f.pdf). Any deviations from the pre-registration are noted. Analyses that were not pre-registered are reported in the Exploratory Analyses sub-section of the Results section. We removed one hypothesis listed in our pre-registration (i.e., that gamers who play first-person shooter games will have faster reaction times but will make a similar number of errors in RT-based tasks, since the sample size of first-person shooter players playing over 10 h per week was too small (*n* = 14) compared with those playing less than three hours per week (*n* = 119). The raw dataset used for the statistical analyses can be accessed online as well on OSF (https://osf.io/s2vcz/?view_only=88caada978f141f787684cc2e63b7673).

## Results

**Figure 1:**
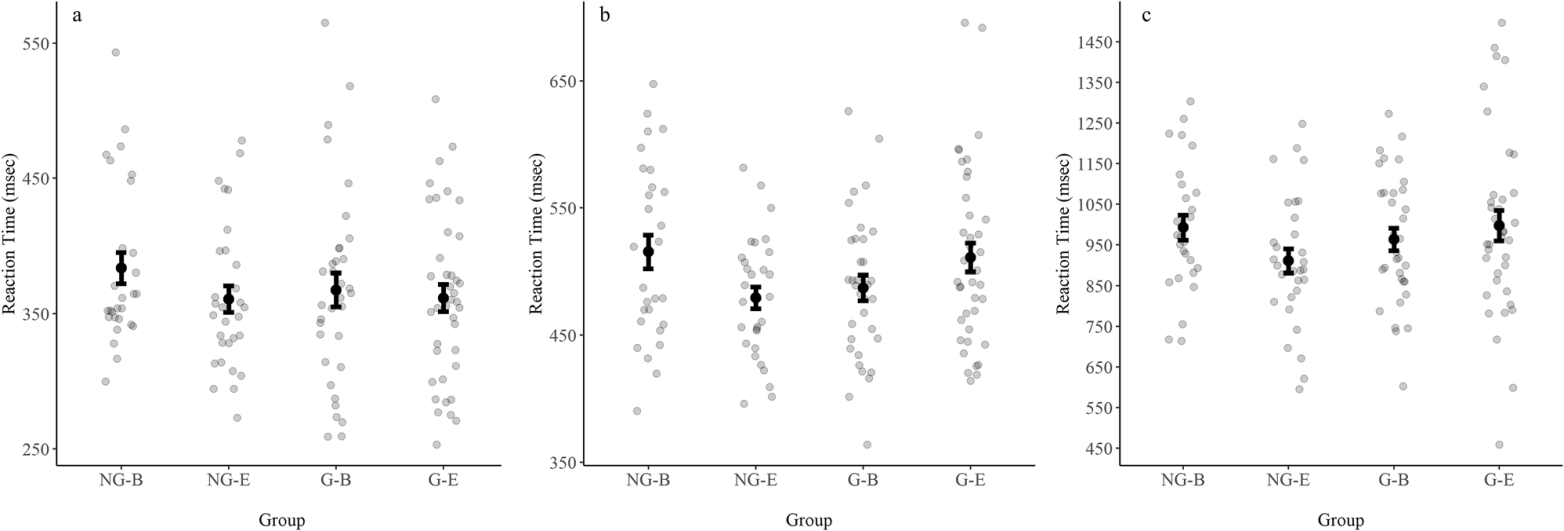
Mean RTs for the choice-RT task (**a**), the Simon task (**b**), and the alternate task-switching task (**c**), for the four experimental groups (figure created using R software). *NG-B* Non-gamers, questionnaire at the beginning, *NG-E* Non-gamers, questionnaire at the end, *G-B* Gamers, questionnaire at the beginning, *G-E* Gamers, questionnaire at the end. Note that the y-axis limits differ between graphs. Errors bars represent standard error. Small light-gray circles represent individual participants.

The results are reported for each of the experimental tasks separately. The RT data for all three RT-based tasks are presented in Fig. 1.

### Choice-RT task

#### Mean RT

A two-way ANOVA [Group × Questionnaire Timing (before or after the tasks)] revealed no group effect, *F*(1, 127) = 0.47, *p* = 0.49, $\eta_{p}^{2}$ = 0.00 and no Questionnaire Timing effect, *F*(1, 127) = 1.68, *p* = 0.20, $\eta_{p}^{2}$ = 0.01. In addition, no significant interaction was found, *F*(1, 127) = 0.59, *p* = 0.44, $\eta_{p}^{2}$ = 0.01. The mean choice RT was 367.72 ± 62.73 ms.

#### Mean correct responses

There were no differences between questionnaire delivery time (before or after the task) in gamers (Mann–Whitney U = 638.00, *p* = 0.74; mean: 23.73 ± 0.41) and non-gamers (Mann–Whitney U = 358.50, *p* = 0.26; mean: 23.71 ± 0.53). There were also no differences in total correct responses between gamers and non-gamers (Mann–Whitney U = 2172, *p* = 0.76).

### Simon task

#### Mean RT

**Figure 2:**
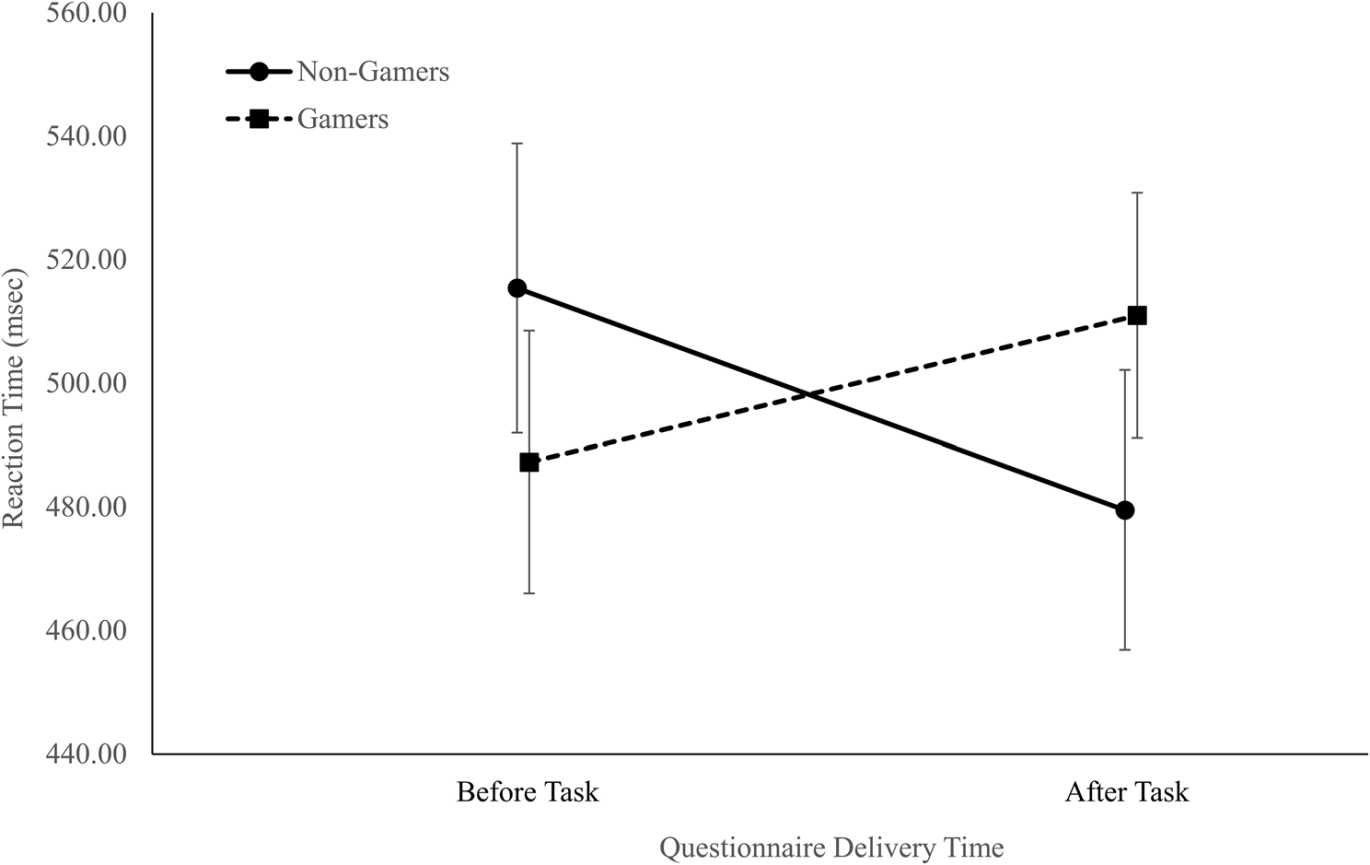
The interaction between group (gamers vs. non-gamers) and the questionnaire delivery time (before vs. after the task) of the mean RT during the Simon task (error bars represent 95% confidence intervals) (figure created using Microsoft Excel).

A two-way ANOVA [Group × Questionnaire Timing (before or after the tasks)] revealed a significant interaction, *F*(1, 127) = 7.30, *p* = 0.01, $\eta_{p}^{2}$ = 0.05, as can be seen in Fig. 2. The mean RT of the non-gamers was higher when the questionnaire was delivered before performing the task (515.40 ± 70.26 ms) compared with after the task (479.51 ± 47.57 ms; Cohen’s *d* = 0.61). In contrast, the mean RT of gamers was lower when the questionnaire was delivered before the task (487.26 ± 57.75 ms) compared with after the task (510.98 ± 70.57 ms, Cohen’s *d* = 0.37). There was no Group effect, *F*(1, 127) = 0.02, *p* = 0.88, $\eta_{p}^{2}$ = 0.00, and no Questionnaire Timing effect, *F*(1, 127) = 0.30, *p* = 0.58, $\eta_{p}^{2}$ = 0.00.

#### Mean correct responses

There were no differences in the questionnaire delivery time (before or after the task) between gamers (Mann–Whitney U = 608.00, *p* = 0.66; mean: 22.60 ± 1.32) and non-gamers (Mann–Whitney U = 404.50, *p* = 0.81; mean: 22.35 ± 1.41). There were also no differences in total correct responses between gamers and non-gamers (Mann–Whitney U = 2309.50, *p* = 0.29).

### Alternate task-switching task

#### Mean RT

A two-way ANOVA [Group × Questionnaire Timing (before or after tasks)] revealed no group effect, *F*(1, 123) = 0.77, *p* = 0.38, $\eta_{p}^{2}$ = 0.01, no Questionnaire Timing effect, *F*(1, 123) = 0.53, *p* = 0.47, $\eta_{p}^{2}$ = 0.00, and no interaction, *F*(1, 123) = 3.12, *p* = 0.08, $\eta_{p}^{2}$ = 0.03. The mean RT for this task was 967.43 ± 184.01 ms.

#### Mean correct responses

There were no differences in the questionnaire delivery time (before or after the task) between gamers (Mann–Whitney U = 495.50, *p* = 0.83; mean: 21.93 ± 2.01) and non-gamers (Mann–Whitney U = 361.00, *p* = 0.79; mean: 21.42 ± 2.73). There were also no differences in total correct responses between gamers and non-gamers (Mann–Whitney U = 1758.00, *p* = 0.77).

### Digit span task

#### Mean correct responses

A two-way ANOVA [Group × Questionnaire Timing (before or after the tasks)] revealed no group effect, *F*(1, 127) = 2.32, *p* = 0.13, $\eta_{p}^{2}$ = 0.02, no Questionnaire Timing effect, *F*(1, 127) = 0.22, *p* = 0.64, $\eta_{p}^{2}$ = 0.00, and no interaction, *F*(1, 127) = 0.70, *p* = 0.70, $\eta_{p}^{2}$ = 0.00. The mean correct response was 5.88 ± 1.81.

#### Mean highest number of digits before the first error

A two-way ANOVA [Group × Questionnaire Timing (before or after the tasks)] revealed no group effect, *F*(1, 127) = 1.32, *p* = 0.25, $\eta_{p}^{2}$ = 0.0 and no Questionnaire Timing effect, *F*(1, 127) = 0.63, *p* = 0.43, $\eta_{p}^{2}$ = 0.01. In addition, no significant interaction was found, *F*(1, 127) = 0.64, *p* = 0.43, $\eta_{p}^{2}$ = 0.01. The mean highest number of digits before the first error was 6.69 ± 1.93.

### Stepwise multiple regression and LASSO regression analyses

We entered the following independent variables to the regression equations: hours per week playing video games; playing first-person shooter games, strategy games, and role-playing games; years playing video games; beliefs regarding a connection between playing video games and task performance; and, knowledge of media reports on a connection between playing video games and task performance. Table 1 presents the findings for both the stepwise and LASSO regressions. As can be seen in Table 1, regardless of the type of regression used, the models led to a low *R*^2^ of under 0.06.

**Table 1:** Results of stepwise and LASSO regressions (X represents a predicting variable).

Variable	Regression	Hours playing				Years playing	Beliefs of connection between gaming and performance	Knowledge of media reports	*R*^2^
		Video games	First person shooter	Strategy	Role playing				
Choice RT	Stepwise	–	–	–	–	–	–	–	–
	LASSO	–	–	–	–	–	–	–	–
Simon RT	Stepwise	–	–	–	–	–	–	–	–
	LASSO	–	X	X	–	–	X	–	.04
Alternate task RT	Stepwise	–	–	–	–	X	–	–	.03
	LASSO	X	X	–	X	X	X	X	.05
Choice correct responses	Stepwise	–	–	–	–	–	–	–	–
	LASSO	–	–	–	–	X	–	–	0.0
Simon correct responses	Stepwise	–	–	–	–	–	–	–	–
	LASSO	–	–	–	–	X	–	–	0.0
Alternate correct responses	Stepwise	X	X	–	–	–	–	–	.06
	LASSO	X	X	X	–	–	X	–	.06
Digit span correct responses	Stepwise	–	–	–	–	–	X	–	.04
	LASSO	X	–	–	X	–	X	–	.05
Digit span highest correct	Stepwise	–	–	–	–	–	X	–	.04
	LASSO	–	X	–	–	–	X	–	.04

### Exploratory analyses

#### Gender Differences

We did not expect that gender differences would affect our results, and therefore we did not include an analysis such differences in our preregistration. However, we wanted to make sure that this assumption was indeed the case, and thus performed independent t-tests for all dependent variables in order to assess differences between males and females. Our assumption was correct, as all of these tests were statistically insignificant with low effect sizes (see Table 2).

**Table 2:** Differences between the performance of males and females in all the dependent variables.

Variable	Males (n = 103)	Females (n = 27)	t(df)	*p* value	Cohen’s d
Choice-RT Task RT (ms)	363.04 ± 60.38	384.40 ± 70.42	1.58 (128)	.12	0.33
Simon task RT (ms)	495.06 ± 62.37	511.59 ± 69.24	1.20 (128)	.23	0.25
Alternate task switching RT (ms)	963.70 ± 178.07 (n = 100)	983.16 ± 211.46 (n = 26)	.48 (124)	.63	0.10
Digit span task correct responses	5.98 ± 1.80	5.54 ± 1.85	1.13 (128)	.26	0.24
Digit span task highest # of digits before 1st error	6.72 ± 1.97	6.61 ± 1.86	.27 (128)	.79	0.06

			Mann–Whitney U		
Choice-RT mean correct responses	23.74 ± .40	23.63 ± .72	1399.00	.95	0.23
Simon task RT mean correct responses	22.48 ± 1.37 (n = 102)	22.52 ± 1.35	1393.00	.93	0.04
Alternate task switching mean correct responses	21.53 ± 2.40 (n = 92)	22.25 ± 2.29 (n = 24)	1389.50	.05	0.31

#### Alternate task switching task including all data

**Figure 3:**
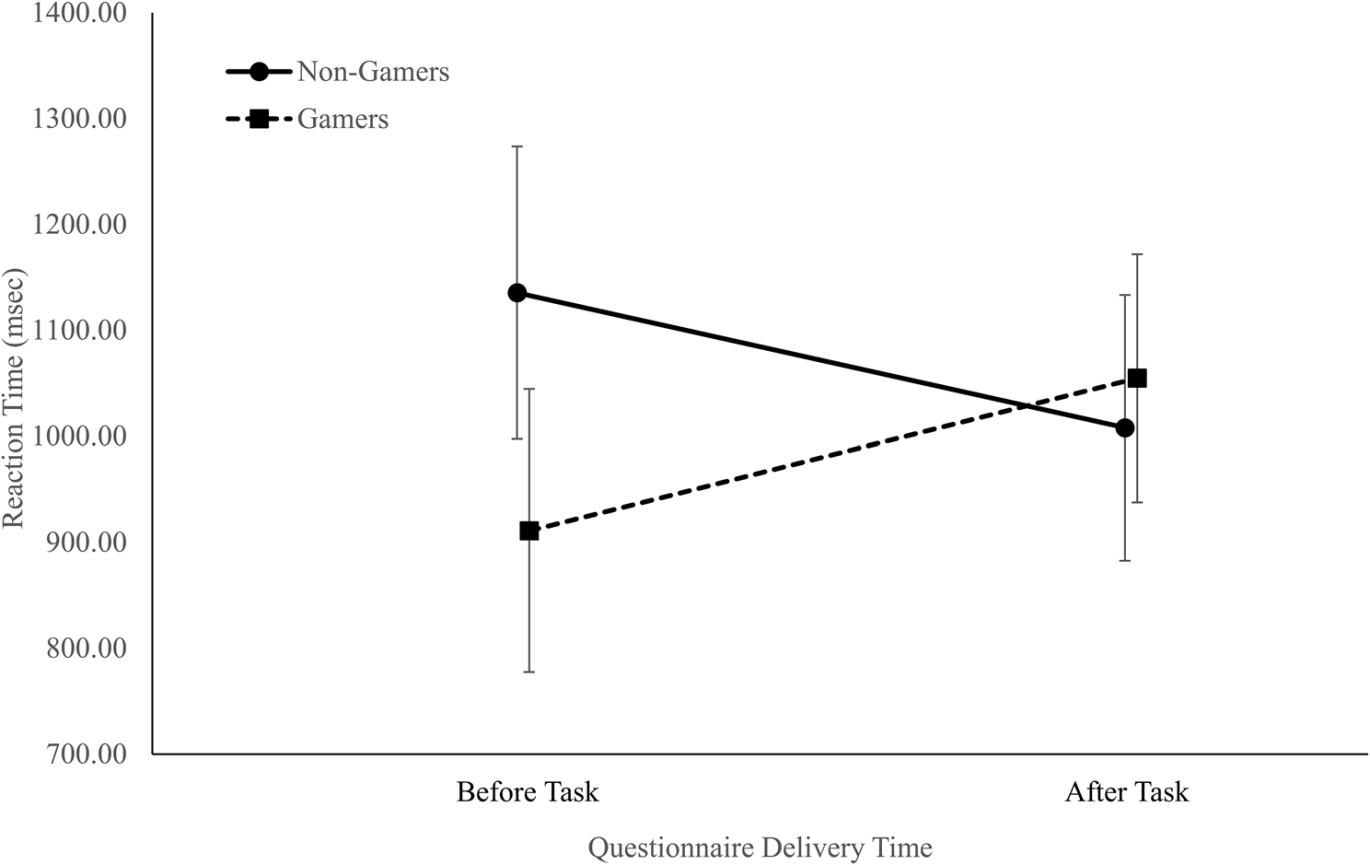
The interaction between group (gamers vs. non-gamers) and the questionnaire delivery time (before vs. after the task) of the mean RT during the alternate task-switching task (error bars represent 95% confidence intervals) (figure created using Microsoft Excel).

For the alternate task-switching task, we decided before the study to remove all RT values over 1500 ms. However, because there was no time limit to the stimulus, durations of over 1500 ms may have been valid as well. Therefore, we ran the two-way ANOVA [Group × Questionnaire Timing (before or after the tasks)] without excluding values over 1500 ms. This analysis revealed a significant interaction, *F*(1, 127) = 4.35, *p* = 0.04, $\eta_{p}^{2}$ = 0.03, as can be observed in Fig. 3. A post-hoc analysis showed that the non-gamers reduced their RT from 1135.45 ± 605.75 ms when the questionnaire was completed before performing the tasks to 911.01 ± 161.57 ms when the questionnaire was answered after performing the tasks (Cohen’s *d* = 0.51). In contrast, the gamers had similar RTs in the beginning questionnaire (1007.94 ± 272.63 ms) and the end questionnaire (1054.83 ± 332.94 ms). There was neither a Group effect, *F*(1, 127) = 0.02, *p* = 0.90, $\eta_{p}^{2}$ = 0.00, nor a Questionnaire Timing effect, *F*(1, 127) = 1.86, *p* = 0.18, $\eta_{p}^{2}$ = 0.01.

#### Beliefs about a connection between playing video games and the ability to perform cognitive-motor tasks

An independent t-test revealed no differences between the beliefs of gamers and non-gamers regarding the connection between playing video games and the ability to perform cognitive-motor tasks, *t*(128) = 1.44, *p* = 0.15, Cohen’s *d* = 0.25. The mean response (on a scale of 1–10) was 8.05 ± 1.93 and 7.60 ± 1.64, for gamers and non-gamers, respectively. The median for both groups was eight.

#### Awareness of media reports on the benefits of video games

In non-gamers (< 3 h of play per week), 25 participants reported that they were aware of media reports discussing the benefits of video games in regards to the performance of cognitive-motor tasks, and 32 participants reported that they were not aware of such reports. In gamers (> 10 h of play per week), 43 reported that they were aware and 30 reported that they were not aware of these reports. However, a χ^2^ test revealed no differences between the groups, χ^2^(1) = 2.90, *p* = 0.09, φ = 0.15.

#### Bayesian analyses of null results

In null-hypothesis significance testing, a lack of significance does not allow us to demonstrate the probability of the null hypothesis itself^32^. Therefore, we used Bayesian statistics to assess the probability of the null hypotheses for the dependent variables that did not produce significant main effects or interactions. The Bayes factors supporting the null hypothesis (BF_01_) compared to the possible combinations of main effects and interactions are presented in Table 3.

**Table 3:** Bayes factors (BF_01_) for the relevant main effects and the interactions that were insignificant in null hypothesis significance testing.

Task	Dependent variable	Model	BF_01_
Choice-RT	RT	Group effect	2.69
		Questionnaire timing effect	4.33
		Both effects	11.75
		Both effects + interaction	38.12
Alternate task switching	RT	Group effect	3.44
		Questionnaire timing effect	4.61
		Both effects	15.85
		Both effects + interaction	16.87
Digit span memory	Correct responses	Group effect	1.91
		Questionnaire timing effect	4.79
		Both effects	9.32
		Both effects + interaction	35.28
	Highest number of digits before first error	Group effect	3.08
		Questionnaire timing effect	3.78
		Both effects	11.92
		Both effects + interaction	34.52

We also analyzed the Bayes factors to exclude the interaction effect alone. This analysis showed that the Bayes factors for excluding the interaction were 16.51, 6.3, 15.70, and 14.94 for the choice-RT task RT, the alternate task switching RT, the correct response, and the highest number of digits before first error in the digit-span task, respectively.

## Discussion

The current study examined whether cognitive/motor task performance in gamers and non-gamers was affected by whether they completed a video-games questionnaire prior to performing those tasks. We had three hypotheses. First, we expected that asking gamers about their gaming experience before the study would lead to better performance in RT-based tasks compared with asking the same questions after the study. This hypothesis was partially supported. Gamers had faster RTs when they performed the Simon task (but not the other two RT-based tasks) after answering the video-games questionnaire compared with before answering the questionnaire. The Bayes factors associated with these tasks mostly suggested that the data are more likely to be accurate under the null hypothesis (except for inconclusive findings regarding the models with the separate main effects) (see Table 3). Second, we hypothesized that this effect would not be found in non-gamers. This hypothesis was not supported by our data. In the Simon task, non-gamers had faster RTs when performing the task before completing the questionnaire compared with after answering the questionnaire. In addition, our exploratory analysis showed that this also occurred in the alternate task-switching task. These results suggest that answering a video-games questionnaire before performing such tasks may have an adverse effect on performance in non-gamers. Finally, our hypothesis that similar effects would not be found for the digit-span memory task was supported by the data of the current experiment.

The finding that the timing of questionnaire delivery affects both gamers and non-gamers can explain, at least in part, the observed differences between groups in previous studies in which all participants answered a video-games questionnaire prior to their participation in the study. According to the results obtained in our study, not only do gamers perform better after answering questions about their gaming habits, but non-gamers perform worse after answering such questions. In fact, our data suggest that it is possible that the effect on non-gamers is greater than the opposite effect on gamers, since answering questions about video-games habits negatively affected the non-gamers in two tasks—the Simon task and the alternate task-switching task (although this is an exploratory finding), whereas this only positively affected gamers in the Simon task. Moreover, there were no differences between groups in the participants' responses to the question “Do you think there is a connection between playing video games and the ability to perform cognitive-motor tasks, such as the ones you just performed?”. In both groups, the mean response was ~ 7.5–8 (on a scale of 1—not at all to 10—very much so). In addition, there were no differences between groups in the number of participants who were familiar with media reports or research regarding the benefits of playing video games in relation to the ability to perform cognitive-motor tasks. Both gamers and non-gamers appeared to believe that playing video games can enhance performances of cognitive-motor tasks, and therefore it is possible that the video-games questionnaire caused gamers to perform better and caused non-gamers to perform worse. Therefore, the results of our study support the concept of psychological suggestion as well as the response expectancy theory.

Similar effects can be found in the literature on stereotypes and test performance. These effects suggests that individuals show suboptimal task performance when they know they are "expected to be" weak at that task^33^. The belief that one is supposed to be weak at a task can be due to prior experience, common knowledge, media reports, or a direct manipulation. All those causes are forms of psychological suggestion—a phenomenon in which what individuals are made to believe, think, or feel can influence their cognition and patterns of behavior positively or negatively^19^. For example, Beilock et al.^34^ (Exp. 1) randomly assigned 40 male expert golfers to a stereotype-threatened group or a control group and asked them to putt from three distances. The participants in the stereotype group were told that women tend to perform these putting tasks better than men and that these differences are supported by statistics from the Professional Golf Association and the Ladies Professional Golf Association. While there were no differences in putting performances between groups in a pre-test, golfers in the stereotype-threatened group performed worse in a post-test compared with their counterparts in the control group.

Psychological suggestion can affect, among other factors, the motivation of the participants. Therefore, it is possible that once gamers realized that the study is about gaming, their motivation to perform better was directly elevated. This elevated motivation may have led to behavioral changes that led to the improved performance in gamers when they performed the tasks after answering the video games questionnaire. Regardless of the reason for the elevated motivation, it has been shown that such motivation can increase focus on the task at hand, and therefore leading to improved performance and learning^35^. While the effects of psychological suggestion and/or motivation are plausible mechanisms for improved performance, the actual underlying mechanism are still to be examined directly in additional studies.

In the current study, the effects of the questionnaire timing on performance in both gamers and non-gamers were found only in some of the performed tasks. In the digit-span task, despite previous studies showing improved working memory performance in gamers (e.g., N-back task^15^), we did not expect any differences between gamers and non-gamers in memorizing digits, as this is not usually a beneficial attribute in video games. However, it is possible that working memory may still benefit from video games, as this cognitive function could be facilitated by improvement in attention or processing speed since the brain networks mediating memory functions and executive function appear to overlap^36^. There were no differences between groups and conditions in the choice-RT task as well. We suspect that this is because the task was too easy, and therefore was not sensitive enough to account for the possible priming effect of the questionnaire. The Simon task was of moderate difficulty and presented the greatest effect of questionnaire timing. Finally, the alternate task-switching task, the most challenging of the three RT-based tasks, showed a questionnaire timing effect only for the non-gamers in the exploratory post-hoc analysis. It is possible that task difficulty serves as a moderator for such stereotype effects^37,38^. Barber et al.^37^, for example, showed that negative age-based stereotyping negatively affected the gait of older adults in a difficult gait task but not in a simple gait task. Additional studies that examine the effects of playing video games on generic or practical cognitive-motor performance should address task difficulty as a possible moderator.

Finally, it is possible that our inconsistent and relatively modest findings are because the priming effect itself was subtle as it required participants to indirectly realize that the study is about video gaming (when the questionnaire was introduced prior to the performance of the tasks). It is possible that larger and more consistent effect sizes would have been found if participants were overtly recruited for this study. However, it was our purpose to examine the effects of subtle and indirect cues on performance, and thus we chose covert recruitment and indirect cues. It should also be noted that in psychological research, as Funder and Ozer^39^ suggested, “small effect sizes from large-*N* studies are the most likely to reflect the true state of nature” (p. 164), and that “Smaller effect sizes are not merely worth taking seriously. They are also more believable” (p. 166). The results of our study, taking into account variability in human behavior, the large sample size, and the relatively subtle intervention, are in line with the abovementioned statements.

### Strengths of the current study

The primary strength of the current study is the covert recruitment of participants. The online participant recruitment platform we used (www.prolific.co) allows the researcher to employ many variables to exclude or include participants based on preliminary answers they supplied when they registered on the website (e.g., demographics, health, hobbies). Furthermore, the researcher can exclude participants who had participated in previous studies completed by the researcher. Once a study is published on that website, participants receive a message that they are eligible to participate, but they do not know the criteria for participation. Hence, the participants in the current study did not know that this was a study that examined the relationships between playing video games and cognitive-motor performance, nor did they participate in any of our previous studies in which similar tasks were used. This is a major methodological issue in video-game research^18^, and therefore we believe that our methodology allowed us to provide meaningful answers to our research questions.

Another strength of the current study is the large sample size and ample statistical power. Many of the studies that compared gamers to non-gamers used relatively small sample sizes [e.g., 36 participants^17^, 21 participants^13^, 35 participants^14^]. In the current study, we were able to recruit 131 participants, who provided us with at least 80% of statistical power. Finally, conducting the study online ensured that it was double-blinded. In addition to the covert recruitment, the researchers in such an online study do not have any contact with the participants, and thus cannot influence their performance in any way.

One final strength is the computerized randomization to experimental groups. This randomization is performed without the knowledge or the intervention of the researchers, and therefore prevents bias in assigning participants to groups.

### Limitations of the current study

One limitation of the current study is that the sample size did not include enough participants who were first-person shooter players or action video-game players. In previous studies, it was mainly playing action video games that was associated with improved cognitive-motor performance. However, we would have been required to implement an overt recruitment process of participants to specifically recruit those participants, and that would have prevented us from answering our research questions.

Second, in an online study, variables such as type and size of keyboard, screen size, participants’ motivation, and environmental conditions cannot be controlled. However, all the participants used a computer to complete the experimental tasks and did not use a smartphone or a tablet. In addition, Woods et al.^40^ suggested that large sample sizes in online studies can make up for the relative lack of control.

Third, it is possible that self-selection bias led participants who received an invitation to participate in a study on RT and memory. Such self-selection may create a sample that consider themselves as proficient at such tasks. However, if this was the case, our findings may suggest that both negative and positive priming can lead to differences in performance even in a biased sample of participants who perform such tasks well.

Finally, in order to maintain covert recruitment, we could not ask detailed questions about videogame playing habits prior to the study. Therefore, we have no knowledge of the distribution of playing time over the week. It is possible that some of the participants play mostly on weekends (similar to massed practice) while others distribute their playing time more evenly throughout the week (similar to distributed practice). Massed and distributed practice may affect learning differently (e.g.,^41^), and thus this can be an important moderating variable that should be examined in additional studies on gaming.

## Conclusion

The results of the current study suggest that asking participants about their gaming experience before they perform cognitive-motor tasks can either positively or negatively affect their performance, depending on whether they are gamers or non-gamers. In addition, task difficulty is a probable moderator of these effects. The results obtained in our study have methodological implications for future research that examines the differences between gamers and non-gamers, and for research in video-game training aimed at facilitating cognitive-motor performance. Finally, these findings support the concept of psychological suggestion and the response expectancy theory.

## Methods

### Participants

We used G*Power^42^ to perform a priori power analysis for our two-way analysis of variance (ANOVA) [Group (gamers/non-gamers) × Questionnaire Delivery Time (before/after the performance of tasks comparing)]. To the best of our knowledge, no previous studies have directly examined the effects of suggestion or response expectancy on simple cognitive/motor tasks in gamers and non-gamers. However, there are studies from the motor learning literature showing that enhanced expectancies of success which are caused by providing easy criteria of success (e.g.,^21,43^) or visual illusions that lead to a perceived larger target^44^ can lead to improved performance and learning, with effect sizes varying from moderate (Cohen's *d* = 0.54;^44^ to large (Cohen’s *d* = 0.8^21^, calculated from the reported $\eta_{p}^{2}$ = 0.14). Because these studies had a small sample size (*N* between 36 and 45), effect sizes could have been overestimated (the Winner’s curse^45^. Therefore, in our study we took a more cautious approach and selected a moderate effect size (Cohen’s *d* = 0.5/Cohen’s *f* = 0.25) for our power analysis. We entered this effect size into the power analysis with the following parameters: alpha (two-sided) = 0.05, power = 0.80, allocation ratio 1:1. The results of the power analysis suggested that 128 participants are required to detect differences between groups or to find an interaction with 80% power.

Therefore, our goal was to recruit 128 participants between the ages of 18–35 years and to randomize them to four groups of 32 participants each: (a) gamers who answered a video-games questionnaire at the beginning of the study (G-B), (2) gamers who answered a video-games questionnaire at the end of the study (G-E), (c) non-gamers who answered a video-games questionnaire at the beginning of the study (NG-B), and (d) non-gamers who answered a video-games questionnaire at the end of the study (NG-E).

We recruited participants through Prolific (www.prolific.co)—an online participant database platform that allows the researcher to use various exclusion and inclusion criteria (based on information individuals provide in their profile) and allows the participants to participate in an online study from their own computer.

In such an online study, we cannot know if the participants who begin the study will complete it. Therefore, we recruited 160 participants in two projects. In one project we recruited 80 participants who, according to their information on Prolific, play video games more than 13 h per week (a more stringent criterion than our pre-registered requirement of > 10 h per week), and in another project we recruited 80 participants who play video games less than three hours per week. We were aware of the possibility that the information individuals entered when they created an account on Prolific may not be current, and indeed, out of 159 participants, only 110 matched our pre-registered criteria: 70 participants who reported playing over 10 h per week and 40 participants who reported playing fewer than three hours per week. Therefore, we added 28 participants in another project in order to increase the number of non-gamers. This addition led to a total of 187 participants who completed the study. Out of those, 131 participants (27 females, one participant who did not report gender, mean age = 23.51 ± 4.33 years) matched our gamers and non-gamers inclusion criteria, and they are analyzed in the current study: 34 participants in the G-B group (one female, one unreported gender), 39 participants in the G-E group (seven females), 28 participants in the NG-B group (10 females), and 30 participants in the NG-E group (nine females). It is important to note that the participants were not recruited based on information entered when signing up to our specific study. The participants on Prolific.ac answer general questions regarding demographics, hobbies, health, etc. when joining the database. Based on these data, we were able to filter participants who filled in specific responses. However, the participants did not know why they received an invitation to participate. This allowed us to covertly recruit participants for the study, without them knowing that the study had anything to do with gaming.

Randomization to groups was performed automatically by the web-based platform. Importantly, the prospective participants in Prolific did not know that they were recruited based on their video game playing habits. The participants also reported being fluent in English and were paid 2.5 British Pounds for their participation. The study was approved by the Ethics Committee of The Academic College at Wingate (approval # 303), and all participants filled out an electronic informed consent form on the study’s website prior to their participation. In addition, all methods were performed in accordance with the relevant guidelines and regulations.

### Tasks

Participants were asked to perform the following four tasks.

#### Choice-RT task

In this task, the participants pressed as quickly as possible the “j” key if the word “right” appeared on the right side and the “f” key if the word “left” appeared on the left side of a centralized cross on the computer screen. The words “right” or “left” were presented for 900 ms, followed by 600 ms during which only the centralized cross was displayed^46,47^.

#### Simon task

This task is a variation of the choice-RT task. The words “right” or “left” could be displayed on either the right or the left side of the cross. The participants were required to press the “j” key if they saw the word “right” (even if it appeared on the left side of the cross) and to press the “f” key if they saw the word “left” (even if it appeared on the right side of the cross)^48,49^. Similar to the choice-RT task, the words “right” or “left” were presented for 900 ms, followed by 600 ms during which only the centralized cross was displayed.

#### Alternate task-switching task

**Figure 4:**
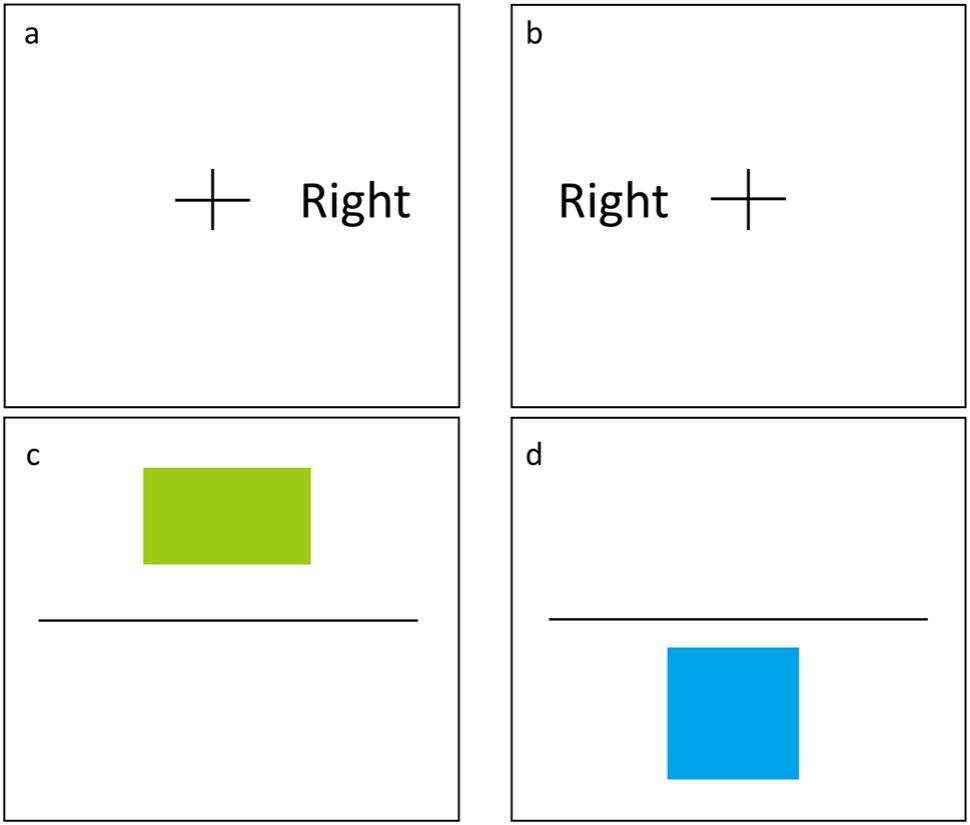
An example of the choice-RT task (**a**), the Simon task (**b**), and the alternate task-switching task (**c**, **d**) (figure created using Microsoft PowerPoint).

In this task, a square or a rectangle in either a blue or green color appeared at the top or at the bottom of the screen. If a shape appeared at the top of the screen, the participants were asked to press the “f” key if the shape was blue and the “j” key if the shape was green (regardless of whether it was a square or a rectangle). However, if the shape appeared at the bottom of the screen, participants were asked to press the “f” key if the shape was a square and the “j” key if the shape was a rectangle (regardless of the color). In this task, each stimulus was presented for an unlimited duration until a key press was recorded. The above-mentioned three RT tasks are presented in Fig. 4.

#### Digit-span memory task

In this task, participants were asked to remember the digits that were presented to them on the screen. The first number included three digits and each consecutive number had one additional digit up to 11 digits. Digits were shown one at a time for a period of one second each. Digits were randomly selected using a random number sampling of the digits 0 to 9 without replacement up to 10 digits. For the 11-digit number an additional (duplicate) digit was randomly added to the 10 digits. All digit randomizations were conducted in R^50^. If the random sample included a series of ascending or descending numbers (e.g., 1, 3, 5; 7, 5, 3; 3, 6, 9; 8, 6, 4) the series was deleted, and another random sample was generated. A similar approach to the presentation of this task has been used in previous experiments (e.g.,^51^).

### Procedure

This study was conducted online using a web-based platform (www.gorilla.sc^52^). This platform is integrated with the participants' database (www.prolific.co) and the participants perform the experiment on their own computer. Web-based studies have been shown to provide accurate measures of RT that are similar to those attained in lab-based studies (e.g.,^53,54^).

After the completion of an electronic informed consent form that was presented at the beginning of the study, participants in the G-B and NG-B groups answered a questionnaire regarding their video game playing habits. Specifically, they were asked how many hours they spend playing video games per week in general, and how many hours they specifically spend playing first-person shooter games, strategy games, or role-playing games. The participants chose one answer from a list (I do not play video games, 1–3 h, 4–6 h, 7–9 h, 10–12 h, 13 h per week or more). In addition, they were asked how many years they have been playing video games (< 1 year, 1–2 years, 3–4 years, 5–6 years, > 7 years). Participants in the G-E and NG-E groups answered a neutral questionnaire with the same number of questions (e.g., how many hours per week do you watch TV, how long is your commute to work, how many books have you read in the last year). After answering the questionnaires, the participants were familiarized with the four tasks in a counterbalanced order. Each participant performed one block of eight trials of the three RT-based tasks (i.e., choice-RT, Simon task, alternate task-switching task) and one block of four trials of the digit span task that consisted of remembering one digit, two digits, three digits, and four digits.

After completing the familiarization stage, the main part of the study began. For the three RT-based tasks, the participants performed two blocks of 24 trials each. For the digit span task, they performed two blocks starting with three digits and ending with 11 digits (in increments of one). The four tasks were presented in a counterbalanced order between participants. After completing the four tasks, participants in the G-E and NG-E answered the same video game playing habits questionnaire. In addition, all four groups answered the following two questions: (a) “Do you think there is a connection between playing video games and the ability to perform cognitive-motor tasks, such as the ones you just performed?” (answers on a scale of 1—not at all, to 10—very much so), and (b) “Are you familiar with media reports or research regarding the benefits of playing video games in relation to the ability to perform cognitive-motor tasks?” (yes or no). These two questions were presented to all groups at the end of the study, because if they were presented at the beginning of the study they could have explicitly exposed the study’s purpose^18^. In all of the questions presented throughout the experiment, the option to answer, “Prefer not to say” was included as well.

### Data exclusion

During pre-registration, we decided that for the choice-RT and the Simon task, RT values of over 1000 ms would be removed because they represented RTs that were longer than the presentation of the stimulus (900 ms). However, this did not occur. For the alternate task-switching task, based on our pre-registration, RT values of over 1500 ms were removed. This resulted in a removal of 17 blocks (out of a total of 262 blocks, 6.5% of the blocks). If there were more than 50% incorrect key presses in a block of 24 trials, the block was deleted, as this most likely shows that the participant did not understand the task. This happened only three times in the Simon task (1.1% of blocks), and eight times in the alternate task-switching task (3.1% of the blocks). During pre-registration, we also decided that if there were over 50% incorrect key presses in both blocks of two of the three RT-based tasks for one participant, this participant would be removed from the study. However, this did not occur with any of the participants.

### Data analyses

For each of the three RT-based tasks we measured RTs (ms) and the number of correct responses. These were averaged for the two blocks of trials in each task. For the digit span task, we measured the maximum number of digits remembered before the first error and the total number of correct answers. These two variables were averaged for the two blocks of trials.

Based on skewness and kurtosis values, RTs were mostly normally distributed and were analyzed using a 2-way ANOVA [Group (gamers/non-gamers) × Timing of questionnaire (before/after tasks)]. The number of correct key presses in the three RT-based tasks was not normally distributed, and because there is no non-parametric equivalent for a two-way ANOVA we used the Mann–Whitney test to examine, for the group of gamers and non-gamers separately, the differences in correct responses between the condition in which the video-games questionnaire was completed before performing the task and the condition in which it was presented after performing the tasks. The variables measured in the digit span memory task were normally distributed and were analyzed using two-way ANOVAs like those used for the analyses of RTs.

In our pre-registration, we wanted to conduct the statistical analyses separately for each type of game played (e.g., general, first-person shooter, strategy games, role-playing). However, the separate sample sizes were too small, and therefore these analyses could not be performed. We also performed a stepwise multiple regression to examine whether video game playing habits and conceptions of the effects of video games on performance could predict RTs and correct responses in the performed tasks. For this analysis only, we used the data of all 187 participants who completed the study. Because stepwise regression can lead to overfitting and over-estimation of models, we also conducted LASSO (Least Absolute Shrinkage and Selection Operator) regression—an accepted alternative to stepwise regression that deals with such problems^55^. To better understand the non-significant effects or interactions, we used Bayesian statistics in our exploratory analyses.

Statistical analyses were conducted using the SPSS version 25 (SPSS Statistics, IBM, USA), R^50^ for LASSO regression, and JASP^56^ for all Bayesian analyses. Bonferroni post-hoc analyses and 95% confidence intervals were used for post-hoc testing when appropriate, and alpha was set at 0.05.

## Data Availability

The raw data for this study is available in a raw data repository: https://osf.io/s2vcz/?view_only=88caada978f141f787684cc2e63b7673. The pre-registration is available here: https://aspredicted.org/wp53f.pdf.
